# Application of Convolutional Neural Networks Using Action Potential Shape for In-Silico Proarrhythmic Risk Assessment

**DOI:** 10.3390/biomedicines11020406

**Published:** 2023-01-30

**Authors:** Da Un Jeong, Yedam Yoo, Aroli Marcellinus, Ki Moo Lim

**Affiliations:** 1 Department of IT Convergence Engineering, Kumoh National Institute of Technology, Gumi 39253, Republic of Korea; 2 Department of Medical IT Convergence Engineering, Kumoh National Institute of Technology, Gumi 39253, Republic of Korea; 3 Meta Heart Inc., Gumi 39253, Republic of Korea

**Keywords:** drug screening, action potential shape, convolutional neural network

## Abstract

This study proposes a convolutional neural network (CNN) model using action potential (AP) shapes as input for proarrhythmic risk assessment, considering the hypothesis that machine-learning features automatically extracted from AP shapes contain more meaningful information than do manually extracted indicators. We used 28 drugs listed in the comprehensive in vitro proarrhythmia assay (CiPA), consisting of eight high-risk, eleven intermediate-risk, and nine low-risk torsadogenic drugs. We performed drug simulations to generate AP shapes using experimental drug data, obtaining 2000 AP shapes per drug. The proposed CNN model was trained to classify the TdP risk into three levels, high-, intermediate-, and low-risk, based on in silico AP shapes generated using 12 drugs. We then evaluated the performance of the proposed model for 16 drugs. The classification accuracy of the proposed CNN model was excellent for high- and low-risk drugs, with AUCs of 0.914 and 0.951, respectively. The model performance for intermediate-risk drugs was good, at 0.814. Our proposed model can accurately assess the TdP risks of drugs from in silico AP shapes, reflecting the pharmacokinetics of ionic currents. We need to secure more drugs for future studies to improve the TdP-risk-assessment robustness.

## 1. Introduction

The International Council for Harmonization (ICH) established the S7B and E14 guidelines for drug safety assessment, which present a human ether-à-go-go (hERG) block and a prolonged QT interval as critical indicators of evaluation based on in vitro and in vivo methods [1,2]. The hERG assay has high sensitivity, allowing high-risk candidates to be rapidly and strictly excluded from drug development. However, as its specificity is low, it discontinues development of potential therapeutic drugs that prolong action potential duration (APD) but do not induce torsade de pointes (TdP), a lethal drug-induced syndrome [3,4,5]. Thirteen advanced medical institutions in seven countries are aware of the limitations of the existing guidelines for drug toxicity assessment and have initiated the comprehensive in vitro proarrhythmia assay (CiPA) project to revise the current guidelines and solve the problems caused by the current strict regulations [5,6].

The CiPA guidelines comprise four nonclinical phases: in vitro assessment of multiple ionic currents, in silico computer modeling, in vivo electrocardiograph (ECG) assessment, and in vitro assessment using stem-cell-derived ventricular cardiomyocytes [5]. The in vitro assessment phase of the CiPA includes both an hERG assay and an in vitro assay for six major ion channels, I_Na_, I_NaL_, I_CaL_, I_Ks_, I_K1_, and I_to_, that directly or indirectly affect action potential (AP) repolarization. The dose–response curves of drugs generated from the voltage clamp of the in vitro assay provide important drug characteristic parameters, such as half-maximal inhibitory concentration (IC50) and slope at IC50 (Hill coefficients). The in silico computer modeling phase quantitatively predicts drug effects from the cellular level to the organ level based on IC50 and the Hill coefficient [7].

Following the CiPA guidelines, several researchers have proposed classification models and in silico biomarkers for drug safety assessments. Li et al. presented qInward, the total amount of charge that flows into cells through I_NaL_ and I_CaL_, as an indicator that can detect drugs with high potential to induce TdP [8]. Dutta et al. recommended a qNet value, which is the total amount of net charges that move through I_NaL_, I_CaL_, I_Kr_, I_Ks_, I_K1_, and I_to_, as a new metric to classify the TdP risks of drugs as high-, intermediate-, and low-risk [9]. Li et al. calculated the qNet value of 28 drugs and assessed their TdP risk using threshold qNet values calculated using hERG-dynamic whole-cell simulation and non-hERG whole simulation [10,11]. The classification accuracies without hERG were 86% for high-risk drugs and 85.6% for low-risk drugs [11], whereas those with hERG dynamics were 98.8% for high-risk drugs and 90.1% for low-risk drugs [10]. Miram et al. suggested a linear discriminant analysis using APD at 90% repolarization (APD_90_), classifying 31 drugs into a five-risk level at an average error rate of 0.323 [12]. Llopis-Lorente et al. presented a decision-tree model using TqNet, the qNet response rate based on drug concentration, and showed 91.7% accuracy in predicting the TdP risks of drugs [13]. Lancaster et al. found remarkable differences in APD at 50% repolarization (APD_50_) and diastolic Ca^2+^ concentrations between torsadogenic and nontorsadogenic drugs. Using these two metrics, a support vector machine (SVM) model successfully identified torsadogenic drugs with 96.3 % accuracy and a 12.8% misclassification rate [14].

It is essential to select significant input indicators in machine-learning models for TdP risk assessment [7]. There have been many attempts to select the most accurate and reasonable input indicators for drug classification [9,15]. The aforementioned studies have suggested various physiological biomarkers, but the selection criteria thereof remain unclear, and many covered potential biomarkers may still exist. We speculate that raw AP shape has more potential characteristics than only one or two biomarkers. Therefore, this study proposes a convolutional neural network (CNN) model using AP shapes as input for proarrhythmic risk assessment, with the hypothesis that the machine-learning features automatically extracted from AP shapes contain more meaningful information than do manually extracted indicators. The proposed CNN model classifies the TdP risk of CiPA drugs into three levels, high-, intermediate-, and low-risk, based on the criteria described by Li et al. [10].

## 2. Materials and Methods

### 2.1. Drug Experimental Data

We used the 28 drugs listed by Li et al., opened in the GitHub. They included eight high-risk, eleven intermediate-risk, and nine low-risk torsadogenic drugs (Table 1) [10]. The CiPA experimental data had block percentages measured through a voltage clamp in seven ion channels, I_Na_, I_NaL_, I_Kr_, I_Ks_, I_K1_, I_to_, and I_CaL_, according to four variations of drug concentration (https://github.com/FDA/CiPA (accessed on 28 September 2022)). We preprocessed these in vitro data following the methodology of Crumb et al. [11]. First, the experimental data were bootstrapped to quantify uncertainty using the Markov Monte Carlo (MCMC) method proposed by Chang et al. [16], and the half-maximal inhibitory concentration (IC50) and slope coefficients at IC50 (hill coefficient) of 2000 hill curves within a 95% confidence interval were computed. The in silico model used these IC50 and Hill coefficients to implement static drug binding for ion channels and determined AP, reflecting the drug effects (see the Section 2.2). 

### 2.2. In Silico Model

**Figure 1:**
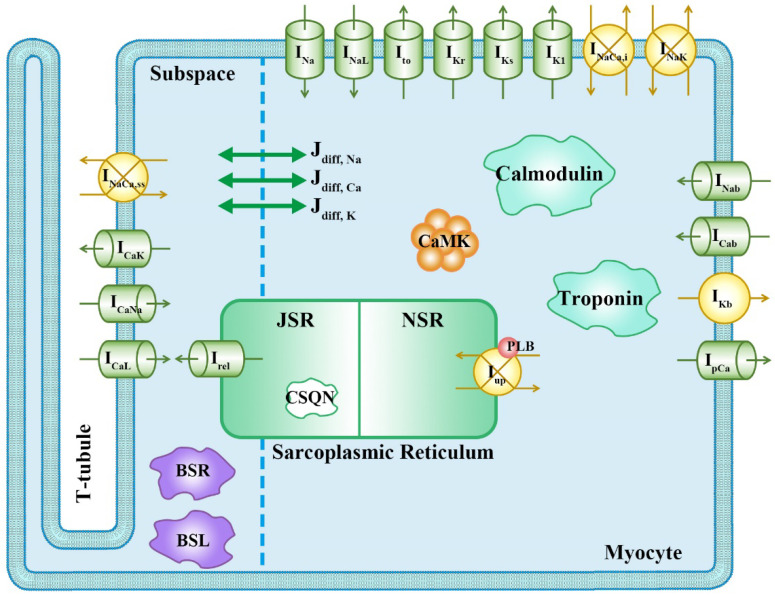
Schematic of in silico cell model for drug simulation; the ventricular cell model used in this study is the ORd model optimized to assess drug effects as described by Dutta et al. I_Na_, fast Na^+^ current; I_NaL_, L-type Na^+^ current; I_to_, transient outward K^+^ current; I_Kr_, rapid-delayed rectifier K^+^ current; I_Ks_, slow-delayed rectifier K^+^ current; I_K1_, inward rectifier K^+^ current; I_NaCa, i_, 80% of Na^+^-Ca^2+^ exchange current; I_NaCa, ss_, 20% of Na^+^-Ca^2+^ exchange current; I_NaK_, Na^+^-K^+^ exchange current; I_CaK_, Ca^2+^-K^+^ exchange current; I_CaNa_, Ca^2+^-Na^+^ exchange current; I_CaL_, L-type Ca^2+^ current; I_Nab_, background Na^+^ current; I_Cab_, background Ca^2+^ current; I_Kb_, background K^+^ current; I_pCa_, Ca^2+^ pump current; J_up_, Ca^2+^ upstroke flux from myocyte into network sarcoplasmic reticulum (NSR); J_rel_, Ca^2+^ flux through ryanodine receptor inside junctional sarcoplasmic reticulum (JSR); J_diff, Na_, Na^+^ diffusion flux between subspace and myoplasm; J_diff, Ca_, Ca^2+^ diffusion flux between subspace and myoplasm; J_diff, K_, K^+^ diffusion flux between subspace and myoplasm; PLB, phospholamban; CSQN, calsequestrin; CaMK, Ca^2+^-calmodulin-dependent protein kinase II; BSR, anionic SR binding sites for Ca^2+^; BSL, anionic sarcolemmal binding sites for Ca^2+^.

The software for the in silico simulation was written in the C++ language and based on the O’Hara–Rudy (ORd) model that was optimized to assess drug effects, as described by Dutta et al. [9,17]. It has scaled conductances in five major ionic currents: I_Kr_, I_Ks_, I_K1_, I_CaL_, and I_NaL_ (Figure 1). The ionic current blocked by the drug was expressed through the Hodgkin–Huxley equation, multiplied by the inhibition factor (IF) equation that consists of the *IC*50, the Hill coefficient (h), and the drug concentration (*D*), as shown in Equations (1) and (2):

(1) $$\mathrm{inhibition}\;\mathrm{factor}\;\left(\mathrm{IF}\right)=\frac{1}{1+{\left(\frac{IC50}{\left[D\right]}\right)}^{h}}$$

(2) $$I_{\mathrm{ion}}=\mathrm{IF}\cdot G_{\mathrm{ion}}\cdot m_{\mathrm{ion}}\left(V-E_{\mathrm{ion}}\right)$$

where I_ion_ represents the ionic currents inhibited by the drug and G_ion_ is the conductance of specific ionic currents. m_ion_ denotes the gate-state variable and can have more than one value depending on the type of ionic current. V is the membrane potential and E_ion_ is the equilibrium potential of the specific ionic current. Based on this simple form of the ionic-current model, the ORd model comprises complex ionic-current equations with several constants. In the drug simulation, we set 1, 2, 3, and 4 times the peak serum concentration (Cmax) of each drug. All drug simulations used the state values of the gates and currents that reached steady-state conditions after 10,000 pacings without drug effects as initial cell-model values [9]. These stimulations were applied 1000 times at a cycle length of 2000 ms to mimic the bradycardia condition and assess the TdP-inducible risk of a drug via setting the slow pacing cycle of a 30 bpm heart rate, generating 1000 AP beats. The time resolution for this calculation was 0.1 ms and the time resolution for writing the AP profile was 2 ms. Since the beginning of the beating was transient and unstable, we selected AP shapes as the input of the machine-learning model when the slope during the repolarization phase was maximal among 750–1000 pacings (last 250 beats), in which we could capture AP shapes when early after-depolarization (EAD) occurred in a steady state [9]. As we mentioned above, we bootstrapped in vitro experimental data into 2000 samples of IC50 and H per drug using the uncertainty quantification algorithm based on the Markov chain Monte Carlo (MCMC) method and finally obtained a total of 56,000 samples (2000 samples per 28 drugs). When the in silico simulation was performed with drug effects, using the bootstrapped drug samples as inputs, 8000 AP shapes per drug (2000 AP shapes × 4 concentrations) were generated. Then, we randomly extracted 500 APs from these 8000 APs per drug, generating 6000 AP shapes (500 AP shapes × 12 training drugs) total for training the model. These AP shapes were fed into the CNN model as input to classify the proarrhythmic risks of drugs without additional preprocessing, such as filtering, because AP shapes do not have any noise or artifact as computational output of the in silico simulation.

### 2.3. Model Structure

**Figure 2:**
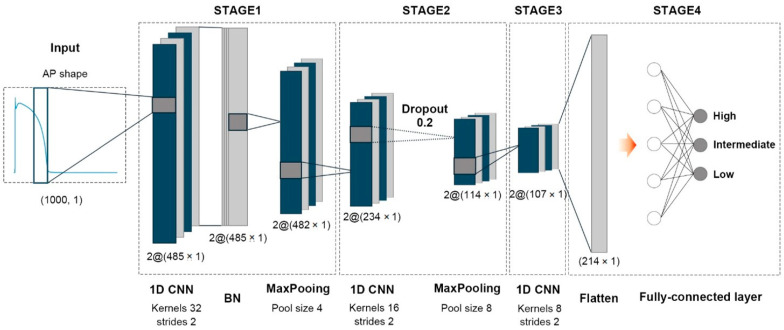
Model structure of proposed CNN model using AP shapes as input: 1D CNN, one-dimensional convolutional neural network layer; BN, batch-normalization layer; AP, action potential. Each AP shape has 1000 data points with a 2 ms time resolution (cycle length of 2000 ms). After passing through three CNN groups, machine-learning features of 214 were extracted and fed into the artificial neural network layers with five nodes.

The proposed CNN model using AP shapes as an input is structured as shown in Figure 2. The input of the CNN model, each AP shape, had 1000 data points with a time resolution of 2 ms. The proposed model comprises three convolutional layers with two filters each, with filter sizes of 32, 16, and 8 for the first, second, and third layers, respectively. The first convolutional layer moved every two strides for the input shape, resulting in an output size of 485. It was connected to the batch-normalization (BN) layer and a max-pooling layer with a size of four to prevent overfitting to the training set. The second convolutional layer was connected to a max-pooling layer with a size of eight for every two strides after passing the drop-out layer at a 20% rate. With the outputs of the last convolutional layer flattened, the 214 generated machine-learning features fed into the hidden layer with five nodes to predict proarrhythmic risk in three levels: high-, intermediate-, and low-risk. Aside from the output layer using the “softmax” activation function, the hidden layer and convolutional layers used the “Rectified Linear Unit (ReLU)” activation function. The loss function for training the model was “categorical cross-entropy,” and the optimization function was “adam” with a learning rate of 0.01.

### 2.4. Model Training and Testing

We used 12 CiPA training drugs to train the model (quinidine, sotalol, dofetilide, bepridil, cisapride, terfenadine, chlorpromazine, ondansetron, verapamil, ranolazine, diltiazem, and mexiletine). As the number of AP shapes for each drug was 2000, the total number of AP shapes for training was 24,000. The model training worked out 100 epochs through 10-fold cross-validation to determine the optimal model with hyperparameters for assessing proarrhythmic risks of drugs. Then, we determined the hyperparameters of the final model through comparison of the classification performances from the validation sets to the training sets, which were randomly distinguished from 12 training drugs. The final model was validated using 16 CiPA test drugs: disopyramide, ibutilide, vandetanib, azimilide, clarithromycin, clozapine, domperidone, droperidol, pimozide, risperidone, asemizole, metoprolol, nifedipine, nitrendipine, tamoxifen, and loratadine. The number of AP shapes for the test was 32,000.

The proposed model was validated through a 10,000-times-repeated testing method in which we randomly extracted samples from each drug set, generating 10,000 test sets [8]. We then plotted the receiver operating curves for 10,000 test sets and evaluated model performance via calculating the area under the curve (AUC), sensitivity, specificity, and likelihood (LR) values. Each value was calculated for individual TdP-risk categories based on the 10,000 AUCs;

(3) Sensitivity=TP/(TP+FN)

(4) Specificity=TN/(TN+FP)

(5) $$\mathrm{Positive}\;\mathrm{likelihood}\;\mathrm{ratio}\;\left(\mathrm{LR}+\right)=\frac{\mathrm{sensitivity}}{1-\mathrm{specificity}}$$

(6) $$\mathrm{Negative}\;\mathrm{likelihood}\;\mathrm{ratio}\;\left(\mathrm{LR}-\right)=\frac{1-\mathrm{sensitivity}}{\mathrm{specificity}}$$

where TP and TN are true positives and true negatives, respectively, indicating that the model correctly answers actual positive and negative problems. An FP is a “false positive”, indicating that the model mispredicts an actual negative problem as positive. An FN is a “false negative”, representing the mispredicted case of an actual positive problem as negative. In the calculation of LR+, we set a small number, close to zero, in the denominator to prevent the result from becoming infinite.

## 3. Results

### 3.1. In Silico Simulation Results

**Figure 3:**
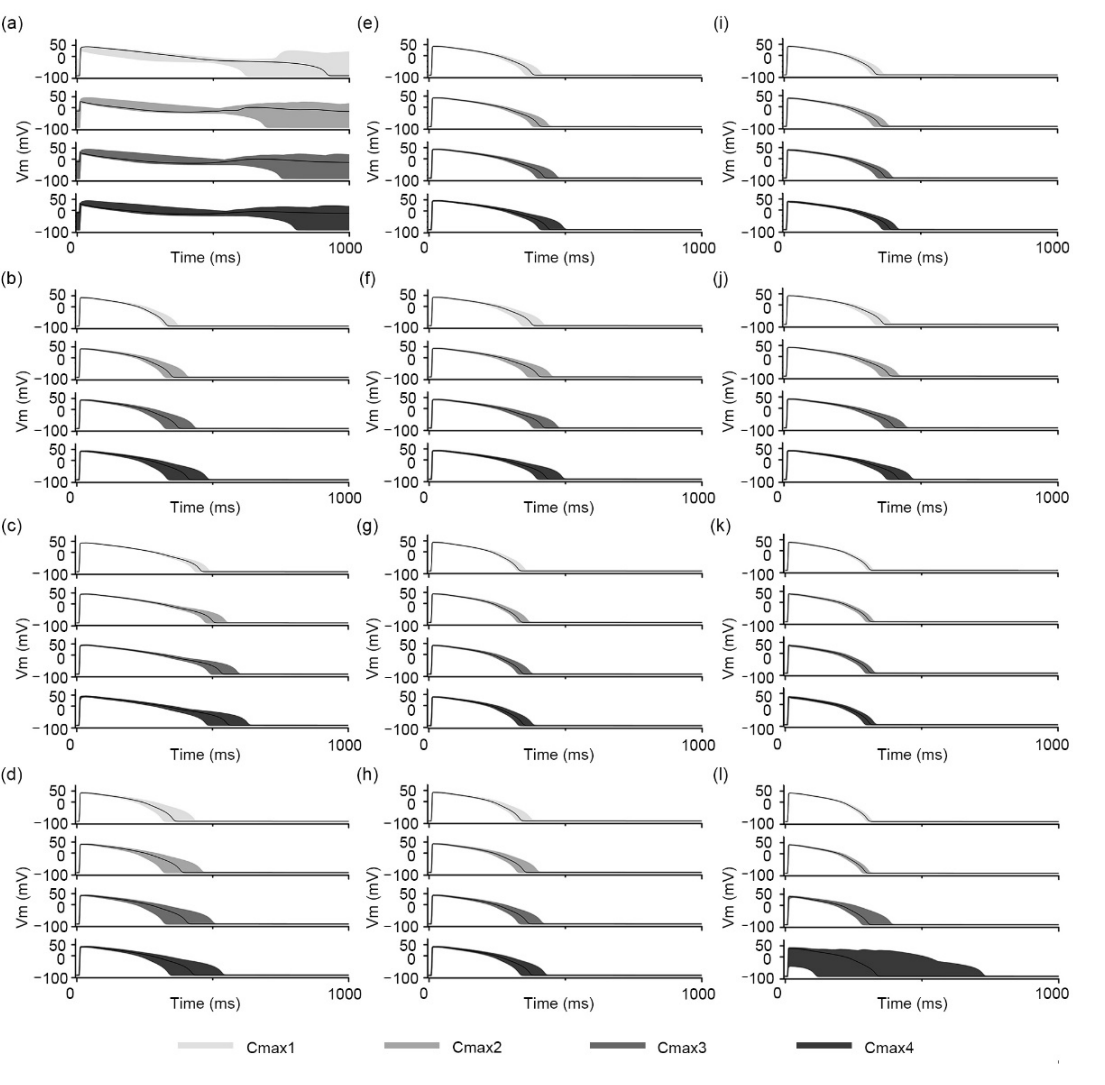
AP traces for 12 training drugs according to the Cmax variation: (**a**) quinidine, (**b**) sotalol, (**c**) dofetilide, (**d**) bepridil, (**e**) cisapride, (**f**) terfenadine, (**g**) chlorpromazine, (**h**) ondansetron, (**i**) verapamil, (**j**) ranolazine, (**k**) diltiazem, and (**l**) mexiletine. The solid black lines denote the median value among 500 AP traces in each Cmax condition.

**Figure 4:**
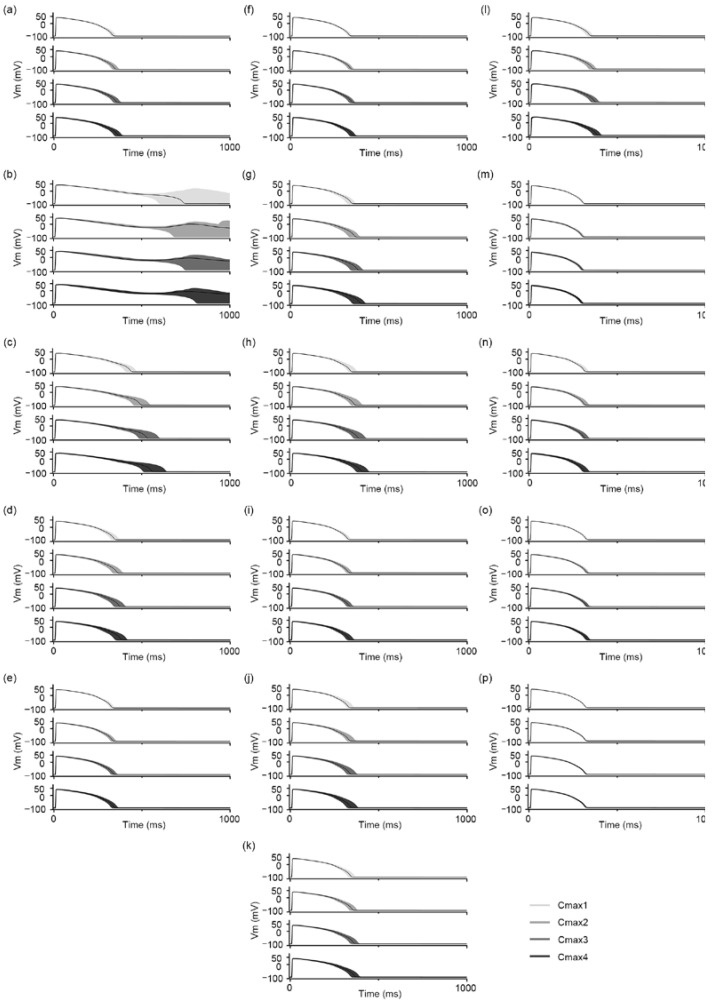
AP traces for 16 test drugs according to Cmax variation: (**a**) disopyramide, (**b**) ibutilide, (**c**) vandetanib, (**d**) azimilide, (**e**) clarithromycin, (**f**) clozapine, (**g**) domperidone, (**h**) droperidol, (**i**) pimozide, (**j**) risperidone, (**k**) astemizole, (**l**) metoprolol, (**m**) nifedipine, (**n**) nitrendipine, (**o**) tamoxifen, and (**p**) loratadine. The solid black lines denote the median value among 500 AP traces in each Cmax condition.

The AP traces of the in silico simulations that observed the effects of drugs according to four Cmax variations are shown in Figure 3 and Figure 4. Figure 3 shows the AP traces of 12 training drugs: (a–d) for high risk, quinidine, sotalol, dofetilide, and bepridil; (e–h) for intermediate risk, cisapride, terfenadine, chlorpromazine, and ondansetron; and (i–l) for low risk, verapamil, ranolazine, diltiazem, and mexiletine. APD_90_ was prolonged as Cmax increased for most drugs, but in the case of diltiazem, the APD_90_ median was not remarkably changed, at 295.4 (295.1–295.7) ms. However, this did not show APD_90_ tendency according to proarrhythmic risk; there was no significant difference in the APD_90_ values between the three categorized risk levels. All of the drugs made the APD_90_ change range wide according to Cmax variation. The APD_90_ variation for Cmax changes was the smallest for diltiazem, at 29.2 (20.4–35.8) ms, followed by that of chlorpromazine, at 43.4 (30.5–53.7) ms. Among the training drugs, EAD was only observed in all Cmax-variation conditions of quinidine. Even though mexiletine is categorized as low-risk, its AP traces were unstable under the Cmax3 (107.4 ms) and Cmax4 (612.9 ms) conditions as compared to other drugs. Accordingly, the change in APD_90_ was the largest for mexiletine, at 190.9 (17.9–612.9) ms, followed by that of bepridil, at 157.3 (195.1–114.0) ms. Mexiletine induced unstable APD and prolonged APD more than did bepridil. This was due to the high uncertainty for the in vitro experimental data sets. If an in vitro experiment is performed in a narrow concentration range that cannot cover all dose–response variations, the uncertainty for the in vitro experimental data set becomes increased in the fitting of the progress of the optimal hill curve that explains the reactions of ionic channels according to drug concentration. Therefore, since this in silico simulation used bootstrapped IC50 and Hill coefficients, which were calculated for in vitro experimental data sets through the bootstrap algorithm (Supplementary Figures S1 and S2), for input, when the quantified uncertainty was high, the simulation results also became unstable. Indeed, the bootstrap results and hill curves for mexiletine were very unstable, while bepridil showed stable bootstrapped curves. Thus, the in silico simulation results for mexiletine showed unstable APD and prolonged APD more than those of bepridil.

Figure 4 shows the AP traces of 16 test drugs: (a–d) for high risk, disopyramide, ibutilide, vandetanib, and azimilide; (e–k) for intermediate risk, clarithromycin, clozapine, domperidone, droperidol, pimozide, risperidone, and astemizole; and (l–p) for low risk, metoprolol, nifedipine, nitrendipine, tamoxifen, and loratadine. Among the test drugs, EAD only occurred in one high-risk drug, ibutilide, as with the training drugs. The test drugs had relatively minor changes, according to Cmax variation, compared to the training drugs; indeed, the most significant change in APD_90_ was 97.3 (59.2–128.0) ms in vandetanib. Nifedipine, tamoxifen, and loratadine, of the low-risk drugs, especially did not show remarkable changes in AP traces, according to four Cmax variations; the APD_90_ median and variation were 286.5 (283.5–293.6) ms and 10.3 (10.2–10.6) ms, respectively, for nifedipine; 313.7 (309.9–316.2) ms and 16.0 (10.2–20.4) ms, respectively, for tamoxifen; and 307.2 (307.2–307.2) ms and 0.001 (0.001–0.001) ms, respectively, for loratadine. The notable difference in APD_90_ between the three proarrhythmic risk levels was not observed in the test drug set, nor, likewise, the training set. We noted the APD_90_ values for each drug according to the Cmax variation provided in Supplementary Tables S1 and S2.

### 3.2. Classification Results

The proposed CNN model predicted the proarrhythmic risks of drugs based on AP shapes. Table 2 shows the evaluation scores for the 95% confidence interval of 10,000 tests. The classification accuracies of the proposed CNN model were excellent for high- and low-risk drugs, with AUCs of 0.914 (confidence range, 0.913–0.916) for high-risk drugs and 0.951 (confidence range, 0.950–0.952) for low-risk drugs. The model performance for intermediate-risk drugs was good, with an AUC of 0.814 in the confidence range of 0.812–0.815. The true negative rate (specificity) of the proposed classifier was 0.853 (0.852–0.855)—0.999 for high risk, 0.664 for intermediate risk, and 0.999 for low risk—and the true positive rate (sensitivity) was 0.70 (0.699–0.702): 0.773 for high risk, 0.853 for intermediate risk, and 0.773 for low risk. The normalized confusion matrix for 10,000 tests of the proposed CNN classifier is shown in Supplementary Figure S3.

The LR+ values of the high- and low-risk drugs were significantly high; the probability that the drugs predicted as high-risk were truly high-risk was higher, at 4465.4 times (confidence interval: 4445.4–4485.3); for low-risk drugs, these values were 1807.4 (1741.3–1873.6) times higher. The probability that the drugs predicted as intermediate-risk were indeed intermediate-risk was only 2.536 (confidence interval of 95%: 2.522–2.550) times higher, which means that the specific AP shape of intermediate risk generated small but sometimes conclusive shifts in the probability of being intermediate-risk. All proarrhythmic risk groups showed similar LR values of 0.2, indicating that assessment of a drug of a specific risk level as the wrong risk level would increase the probability of other risk levels: 0.227 (confidence interval of 95%: 0.225–0.230) for high risk, 0.222 (0.229–0.225) for intermediate risk, and 0.227 (0.225–0.230) for low risk. The proposed CNN classifier is four times (1/LR−) less likely to classify a specific-risk-level drug as the wrong level.

## 4. Discussion

This study proposed a CNN classifier that can reflect the pharmacokinetics of ionic currents using in silico AP shapes to assess the TdP risks of drugs. Many studies have suggested machine-learning models that use AP features, such as APD_90_, APD_50_, APD triangulation (APD_tri_), AP peak voltage, and AP resting, extracted manually from AP shapes [13,14,15]. These features are calculated through feature engineering. Feature engineering, usually used in classical assessment methods, can select significant and objective features with theoretical rationale but make the whole process of assessing drug risk complex. In contrast, the proposed CNN model automatically extracts machine-learning features based on the configuration information of an AP shape according to drug effect, without feature engineering, by working out a random box. Therefore, we guessed that extracted features may have included known information of AP features as well as unknown information, such as temporal correlation, when AP was generated, based on our previous research. Although the physiological meaning of machine-learning features and the exact relationship between features is unknown because the CNN structure works out as a random box, the proposed CNN model can classify drug TdP risks with high accuracy through selection of the most mathematically valuable features. The AUCs of the proposed model were still “excellent” based on the criteria of Han et al., which were 0.9 or higher in the high- and low-risk groups [7].

Llopis-Lorente’s group showed that the accuracy of a decision-tree model with T_qNet as input was 0.917, the true positive rate was 0.90, and the true negative rate was 0.93 [13]. They represented the characteristics of the drug through only one measurement datum, but it could not deal with the uncertainties that could have arisen in in vitro experiments. In this study, the drug data calculated through experimental data were amplified with 2000 IC50 and Hill coefficients so that the drug characteristics could be quantitatively reliable. In addition, drug concentration was expanded to four cases, including the risk of actual drug intake.

In this study, we did not normalize AP shapes before feeding them to the CNN layers, but we added a “Batch Normalization” layer, which normalized each data batch using the mean and standard deviation; here, normalization for training uses the mean and standard deviation of a minibatch, and normalization for testing uses the moving average calculated in the training process. Therefore, even though the distribution of inputs was different, the batch-normalization layer could make zero-mean Gaussian distribution for all data. Additionally, we decided on the model structure through comparison of performance according to various structures, such as batch-normalization number, number of dropout layers, and the position of each layer; then, finally, we added only one batch-normalization layer after the first CNN layer.

The overall strategy for the in silico simulation in this study followed the methodology of Li et al. They assessed the proarrhythmic risks of drugs through logistic regression based on qNet values, suggesting two threshold values that distinguished high- and low-risk drugs from intermediate-risk drugs. They showed a difference in TdP risk-classification performance according to the hERG dynamic considered for drug response [10]. Compared to their qNet logistic regression model, our proposed model especially showed outstanding performance in predicting low-risk drugs, with a 5% higher AUC than that of the hERG qNet logistic regression model, even though we did not consider the hERG dynamic. For high-risk drugs, the classification performance of the proposed model was 7% lower than that of the hERG qNet logistic regression model [10]. This may be because some drugs used in this study may have had ambiguous AP-shape characteristics. Indeed, the AP traces of intermediate-risk drugs were quite different between training and test drugs. Changes according to Cmax variations were more prominent in the intermediate-risk drugs of the training set than those in the test set (Figure 3 and Figure 4). Furthermore, in the study by Li et al., the qNet values of disopyramide, a high-risk drug, were distributed within the intermediate-risk range. We observed that the AP shapes of disopyramide had topological features similar to those of intermediate-risk drugs. To solve this problem, training and validation using various drugs are required.

The proposed model showed lower sensitivity and higher specificity for high- and low-risk drugs. For intermediate-risk drugs, specificity was a bit lower but sensitivity was high. Sensitivity means the ability to accurately predict positive values as positive, whereas specificity refers to the ability to accurately classify negative values as negative; for example, when classifying high-risk drugs (positive values), sensitivity refers to accuracy in detecting those high-risk drugs and specificity refers to the accuracy of classifying intermediate/low-risk drugs. Therefore, low sensitivity and high specificity mean that high-risk drugs and low-risk drugs are more likely to be classified into other risk groups, but not-high-risk drugs and not-low-risk drugs are unlikely to be classified into high-risk groups. 

Our proposed CNN model showed acceptable results for sensitivity and specificity, at over 70%, with minimal levels in screening and diagnostic research [7,18]. To evaluate model performance via combination of sensitivity and specificity, we calculated the LR, denoting the statistical significance of the specific state predicted with a model to the corresponding condition. The proposed model had excellent LR+ values for high and low risk (>10); they were significantly higher than those of the qNet logistic regression with the hERG dynamic (8.05 for high risk and 750,000 for low risk) [10]. The LR values for the three categorized TdP-risk levels were close to the minimum acceptable performance of 0.2. This means that there is a likelihood that the characteristics of drugs classified into the wrong categories will generate small but sometimes essential changes in the probabilities of the corresponding TdP risk categories.

As in this study, the Cai group that used the cardiotoxic risk group classifier with the CNN model used drug molecular structure images, using the molecular operating environment (MOE), as the CNN input without using the results of the in silico model. The characteristics of high sensitivity and low specificity for the classification model remained, and low accuracy was 53.8%–78.1% for drugs that did not block the hERG channel [19]. However, the drug proposed by the CiPA in this study showed high accuracy via mixing 28 drugs with drugs that blocked hERG channels or non-hERG channels.

According to the classification table presented by Han et al., the AUC of the classification model shown was 0.9 or higher in high- and low-risk drugs, which is the highest performance of “excellent.” The LR+ values of the high- and low-risk groups were 10 or higher, which is the highest performance of “excellent.” The performance of the LR indicators was the minimally acceptable performance of 0.2 or higher for the high- and low-risk groups. It should be considered that there is a difference between the existing medical evaluation criteria based on binary classification and the multiple classifications performed in this study, and that the classification performance of the medium-risk group suggested by our classification model could not be directly compared.

This study has several limitations. First, the temporal resolution of the AP shapes was slightly rough, at 2 ms, which might have caused us to miss the upstroke of the membrane voltage. As the CNN model extracts machine-learning features based on the morphological form, its rough temporal resolution may have affected the model’s classification performance. Nevertheless, the proposed CNN model can accurately classify low- and high-risk drugs. Considering the higher AP-shape resolution, we assumed that the CNN model structures would perform better. Second, the TdP-risk labels for the drugs were not standardized. In this study, we followed the methodology described by Li et al., but the labels of drugs can vary according to quantified biomarkers in an experimental data set; Champeroux et al. [20], Woosley et al. [21], and Redfern et al. [22] used different TdP-risk categories based on their results. Finally, the diversity of the proarrhythmic drugs used was insufficient. Even though we generated sufficient drug samples via bootstrapping the in vitro experimental data through the uncertainty quantification algorithm, the total number of drug types was still limited (12) for training the machine-learning model. We thus need to secure more drugs for future studies to improve the robustness of TdP-risk assessments.

## 5. Conclusions

This study proposes a CNN classifier that can automatically extract machine-learning features based on AP-shape configuration information through consideration of the pharmacokinetics of ionic currents from in silico AP shapes in order to assess the TdP risks of drugs. We expect it to be helpful in predicting the cardiac toxicities of new drugs and used as a preliminary validation tool before animal experiments or clinical trials if the proposed methods are validated using more data.

## Figures and Tables

**Table 1:** List of CiPA training and test drugs used in this study [10].

Risk Level	Training Drugs	Test Drugs
High	Bepridil Dofetilide Sotalol Quinidine	Ibutilide Vandetanib Azimilide Disopyramide
Intermediate	Chlorpromazine Cisapride Ondansetron Terfenadine	Domperidone Droperidol Pimozide Astemizole Clopromazine Clarithromycin Risperidone
Low	Verapamil Ranolazine Mexiletine Diltiazem	Nifedipine Nitrendipine Metoprolol Tamoxifen Loratadine

**Table 2:** Classification performances of proposed CNN model. CNN, convolutional neural network; AUC, area under the curve; LR+, positive likelihood; LR−, negative likelihood.

Score	High	Intermediate	Low
AUC	0.914 (0.913–0.916)	0.814 (0.812–0.815)	0.951 (0.950–0.952)
LR+	4465.4 (4445.4–4485.3)	2.536 (2.522–2.550)	1807.4 (1741.3–1873.6)
LR−	0.227 (0.225–0.230)	0.222 (0.219–0.225)	0.227 (0.225–0.230)
Specificity	0.999 (0.999–0.999)	0.664 (0.661–0.667)	0.999 (0.999–0.999)
	0.853 (0.852–0.853)		
Sensitivity	0.773 (0.770–0.775)	0.853 (0.850–0.855)	0.773 (0.770–0.775)
	0.700 (0.699–0.702)		

## Data Availability

The data set of 28 CiPA drugs for this study can be found on GitHub; the data set of Li et al., at https://github.com/FDA/CiPA/tree/Model-Validation-2018 (accessed on 28 September 2022). Aside from in vitro experimental drug data, all of the in silico data were generated through simulations performed by the authors and based on the methods described in this text. The result data used to support the findings of this study are included within this article.

## Supplementary Materials

The following supporting information can be downloaded at: https://www.mdpi.com/article/10.3390/biomedicines11020406/s1, Figure S1. Hill curves of Bepridil for six ionic current channels; Figure S2. Hill curves of Mexiletine for six ionic current channels; Figure S3. Normalized confusion matrix of the 10,000-testing results; Table S1: qNet and APD_90_ according to the Cmax values of training drugs, Table S2: qNet and APD_90_ according to the Cmax values of 16-test drugs.
