# Supplementary material for: Application of Convolutional Neural Networks Using Action Potential Shape for In-Silico Proarrhythmic Risk Assessment
Source: Biomedicines. 2023 Jan 30;11(2):406. doi: 10.3390/biomedicines11020406 (PMC9953470; doi:10.3390/biomedicines11020406)
Supplement: Supplementary file 1 [file biomedicines-11-00406-s001.zip › [MDPI] Supplementary Figures.pdf]

Supplementary Figures

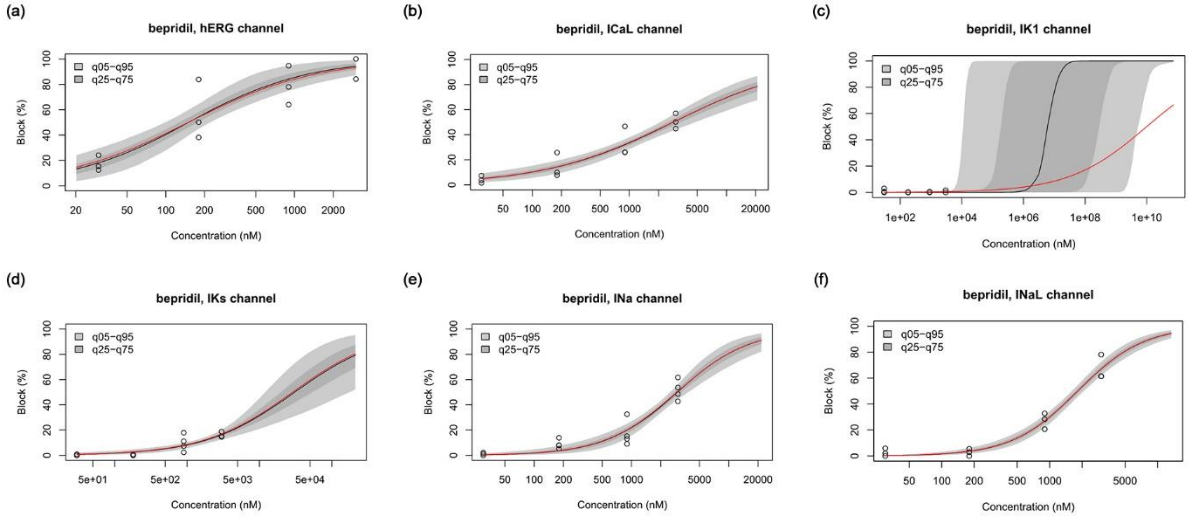

Supplementary Figure S 1. Hill curves of Bepridil for six ionic current channels

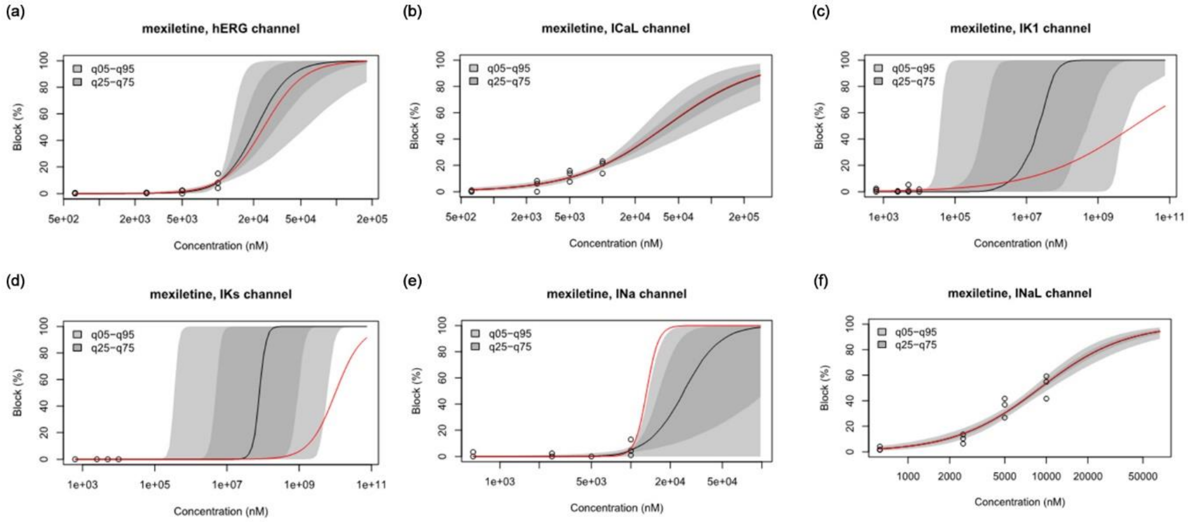

Supplementary Figure S 2. Hill curves of Mexiletine for six ionic current channels

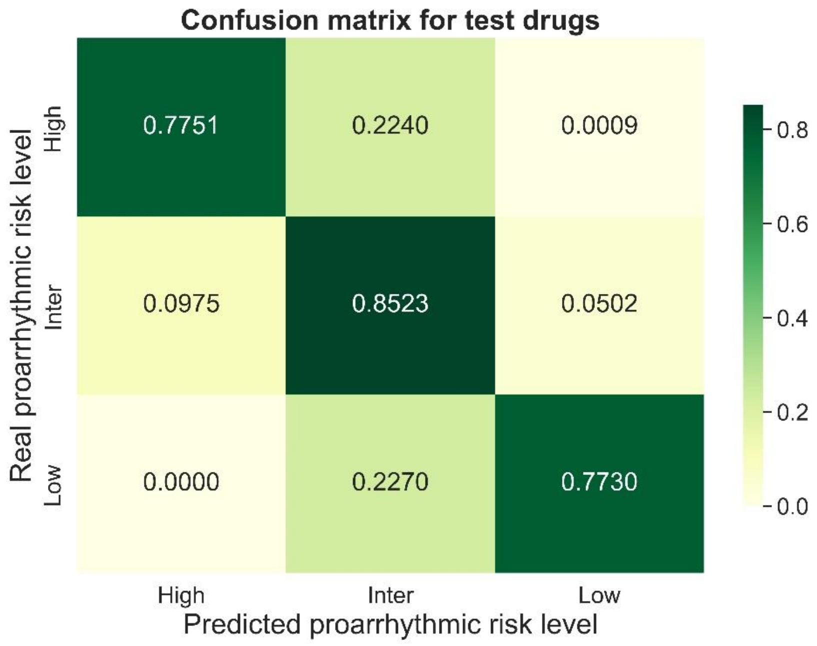

Supplementary Figure S 3. Normalized confusion matrix of the 10,000-testing results
