# Supplementary material for: Application of Convolutional Neural Networks Using Action Potential Shape for In-Silico Proarrhythmic Risk Assessment
Source: Biomedicines. 2023 Jan 30;11(2):406. doi: 10.3390/biomedicines11020406 (PMC9953470; doi:10.3390/biomedicines11020406)
Supplement: Supplementary file 1 [file biomedicines-11-00406-s001.zip › [MDPI] Supplementary table S1.pdf]

Supplementary Table S1. qNet and APD90 according to the Cmax values of training drugs

	class	drug_name	risk_level	cmax	qNet						APD90				
0	training	quinidine	high	3237	Cmax1	Cmax2	Cmax3	Cmax4	Average		Cmax1	Cmax2	Cmax3	Cmax4	Average
				mean	-0.04756	-0.01056	-0.06445	-0.04953	-0.043025	median	904.3233	-	-	-	904.3233
				median	-0.04635	-0.00148	-0.05079	-0.04487	-0.045612	min	602.0953	679.569	736.983	789.292	602.0953
				min	-0.12888	-0.11514	-0.13053	-0.12976	-0.130532	max	-	-	-	-	-
				max	-0.02591	0.019299	0.016883	0.013808	0.019299	max-min	-	-	-	-	-
1	training	bepridil	high	33	Cmax1	Cmax2	Cmax3	Cmax4	Average		Cmax1	Cmax2	Cmax3	Cmax4	Average
				mean	0.044326	0.056956	0.051795	0.047698	0.0501937	median	344.307	371.64	392.483	413.326	382.0615
				median	0.04443	0.056908	0.051633	0.047599	0.049616	min	305.964	304.784	308.127	328.97	304.784
				min	0.031718	0.045727	0.03994	0.03526	0.031718	max	420.012	446.164	486.868	524.032	524.032
				max	0.060109	0.065613	0.066038	0.065172	0.066038	max-min	114.048	141.38	178.741	195.062	157.3078
2	training	dofetilide	high	2	Cmax1	Cmax2	Cmax3	Cmax4	Average		Cmax1	Cmax2	Cmax3	Cmax4	Average
				mean	0.037598	0.030626	0.02653	0.023829	0.0296456	median	444.788	489.031	519.309	542.908	504.17
				median	0.037562	0.030566	0.026516	0.023585	0.028541	min	420.995	459.929	476.053	465.238	420.995
				min	0.033569	0.025208	0.019732	0.010423	0.010423	max	473.89	537.599	579.679	618.613	618.613
				max	0.041207	0.035944	0.033108	0.058825	0.058825	max-min	52.895	77.67	103.626	153.375	96.8915
3	training	sotalol	high	14690	Cmax1	Cmax2	Cmax3	Cmax4	Average		Cmax1	Cmax2	Cmax3	Cmax4	Average
				mean	0.058164	0.053381	0.062234	0.045975	0.0549387	median	317.171	335.655	357.088	397.202	346.3715
				median	0.058399	0.053366	0.062433	0.045874	0.0558825	min	306.356	307.536	308.52	320.515	306.356
				min	0.048595	0.043727	0.055028	0.035916	0.035916	max	354.335	390.32	418.438	465.828	465.828
				max	0.0642	0.06276	0.064527	0.05625	0.064527	max-min	47.979	82.784	109.918	145.313	96.4985
4	training	chlorpromazine	inter	38	Cmax1	Cmax2	Cmax3	Cmax4	Average		Cmax1	Cmax2	Cmax3	Cmax4	Average
				mean	0.061473	0.060755	0.063207	0.062274	0.0619273	median	316.385	321.301	327.396	333.492	324.3485
				median	0.061541	0.060837	0.063309	0.062375	0.0619578	min	306.16	308.52	309.503	313.042	306.16
				min	0.056601	0.055674	0.059365	0.057687	0.055674	max	336.638	349.42	358.071	366.723	366.723
				max	0.065656	0.06568	0.065235	0.065517	0.06568	max-min	30.478	40.9	48.568	53.681	43.40675
5	training	cisapride	inter	2.6	Cmax1	Cmax2	Cmax3	Cmax4	Average		Cmax1	Cmax2	Cmax3	Cmax4	Average
				mean	0.040605	0.051623	0.046646	0.043227	0.0455254	median	365.544	390.124	408.017	424.928	399.0705
				median	0.040665	0.051575	0.046583	0.043233	0.044908	min	337.426	364.561	379.899	391.697	337.426
				min	0.031513	0.046008	0.040613	0.035371	0.031513	max	399.365	425.911	456.783	481.166	481.166
				max	0.045949	0.05742	0.051795	0.047959	0.05742	max-min	61.939	61.35	76.884	89.469	72.4105
6	training	terfenadine	inter	4	Cmax1	Cmax2	Cmax3	Cmax4	Average		Cmax1	Cmax2	Cmax3	Cmax4	Average
				mean	0.045279	0.043062	0.052505	0.048197	0.0472606	median	364.56	387.567	403.887	416.472	395.727
				median	0.045332	0.043166	0.052486	0.048144	0.046738	min	323.66	344.11	365.544	380.684	323.66
				min	0.037211	0.035277	0.044391	0.03993	0.035277	max	405.46	433.383	457.569	475.266	475.266
				max	0.05244	0.049752	0.059528	0.055632	0.059528	max-min	81.8	89.273	92.025	94.582	89.42
7	training	ondansetron	inter	139	Cmax1	Cmax2	Cmax3	Cmax4	Average		Cmax1	Cmax2	Cmax3	Cmax4	Average
				mean	0.061368	0.058742	0.056417	0.054337	0.057716	median	325.43	335.852	349.026	361.611	342.439
				median	0.061537	0.058799	0.056364	0.054251	0.057581	min	308.126	310.289	315.795	323.267	308.126
				min	0.052377	0.04817	0.045442	0.043399	0.043399	max	363.774	384.42	400.151	412.736	412.736
				max	0.065019	0.064488	0.063386	0.061848	0.065019	max-min	55.648	74.131	84.356	89.469	75.901
8	training	diltiazem	low	122	Cmax1	Cmax2	Cmax3	Cmax4	Average		Cmax1	Cmax2	Cmax3	Cmax4	Average
				mean	0.074316	0.077043	0.078565	0.079547	0.0773677	median	295.148	295.379	295.738	295.345	295.362
				median	0.074331	0.077113	0.078619	0.079636	0.0778655	min	284.923	280.597	278.434	277.451	277.451
				min	0.070544	0.072914	0.073644	0.073917	0.070544	max	305.373	308.716	311.076	313.239	313.239
				max	0.077622	0.08073	0.082637	0.084194	0.084194	max-min	20.45	28.119	32.642	35.788	29.24975
9	training	mexiletine	low	4129	Cmax1	Cmax2	Cmax3	Cmax4	Average		Cmax1	Cmax2	Cmax3	Cmax4	Average
				mean	0.090527	0.080027	0.078298	0.08916	0.084503	median	290.429	280.401	291.216	320.318	290.8225
				median	0.09133	0.084658	0.078266	0.089123	0.08689	min	282.76	267.62	265.653	98.907	98.907
				min	0.07428	0.029776	0.074219	0.084028	0.029776	max	300.654	293.182	373.016	711.817	711.817
				max	0.1019	0.106533	0.082785	0.095506	0.106533	max-min	17.894	25.562	107.363	612.91	190.9323
10	training	ranolazine	low	1948.2	Cmax1	Cmax2	Cmax3	Cmax4	Average		Cmax1	Cmax2	Cmax3	Cmax4	Average
				mean	0.063273	0.063839	0.064386	0.064772	0.0640675	median	346.47	371.246	387.764	401.921	379.505
				median	0.063253	0.063887	0.064436	0.06472	0.0641613	min	315.795	335.459	351.976	358.465	315.795
				min	0.057189	0.057614	0.056927	0.055733	0.055733	max	373.803	401.921	428.664	450.49	450.49
				max	0.070695	0.071565	0.071484	0.072447	0.072447	max-min	58.008	66.462	76.688	92.025	73.29575
11	training	verapamil	low	81	Cmax1	Cmax2	Cmax3	Cmax4	Average		Cmax1	Cmax2	Cmax3	Cmax4	Average
				mean	0.063016	0.061071	0.059033	0.064472	0.061898	median	320.515	338.408	356.302	371.836	347.355
				median	0.062895	0.060857	0.058782	0.064413	0.0618758	min	305.177	311.666	325.627	343.718	305.177
				min	0.058891	0.056179	0.053796	0.060805	0.053796	max	348.633	366.527	379.308	399.955	399.955
				max	0.069457	0.06863	0.067237	0.06914	0.069457	max-min	43.456	54.861	53.681	56.237	52.05875
