# Supplementary material for: Application of Convolutional Neural Networks Using Action Potential Shape for In-Silico Proarrhythmic Risk Assessment
Source: Biomedicines. 2023 Jan 30;11(2):406. doi: 10.3390/biomedicines11020406 (PMC9953470; doi:10.3390/biomedicines11020406)
Supplement: Supplementary file 1 [file biomedicines-11-00406-s001.zip › [MDPI] Supplementary table S2.pdf]

Supplementary Table S2. qNet and APD90 according to the Cmax values of 16-test drugs

	class	drug_name	risk_level	cmax	qNet						APD90				
0	validation	ibutilide	high	100	Cmax1	Cmax2	Cmax3	Cmax4	Average		Cmax1	Cmax2	Cmax3	Cmax4	Average
				mean	0.003938	-0.01852	-0.02148	-0.02367	-0.014934	median	724.796	-8.764	-8.764	-8.961	-8.764
				median	0.007265	-0.02007	-0.01971	-0.02004	-0.019877	min	584.792	667.379	730.302	796.174	584.792
				min	-0.028	-0.10518	-0.1056	-0.10579	-0.105786	max	-	-	-	-	-
				max	0.01707	0.008545	0.003171	-0.01873	0.01707	max-min	-	-	-	-	-
1	validation	azimilide	high	70	Cmax1	Cmax2	Cmax3	Cmax4	Average		Cmax1	Cmax2	Cmax3	Cmax4	Average
				mean	0.056593	0.054269	0.052327	0.059554	0.0556857	median	332.116	345.291	359.055	369.28	352.173
				median	0.056619	0.054221	0.05229	0.059564	0.0554198	min	314.223	319.728	328.38	336.049	314.223
				min	0.051735	0.04927	0.047355	0.055297	0.047355	max	355.123	372.82	387.174	399.955	399.955
				max	0.061659	0.059668	0.057707	0.063574	0.063574	max-min	40.9	53.092	58.794	63.906	54.173
2	validation	disopyramide	high	742	Cmax1	Cmax2	Cmax3	Cmax4	Average		Cmax1	Cmax2	Cmax3	Cmax4	Average
				mean	0.059816	0.057988	0.056358	0.061922	0.0590213	median	322.678	331.919	324.447	351.78	328.183
				median	0.059874	0.058081	0.056411	0.061978	0.0589775	min	312.453	319.138	342.341	331.33	312.453
				min	0.05642	0.054498	0.052719	0.05895	0.052719	max	335.459	352.369	362.791	372.23	372.23
				max	0.062913	0.061717	0.060488	0.064259	0.064259	max-min	23.006	33.231	20.45	40.9	29.39675
3	validation	vandetanib	high	255.4	Cmax1	Cmax2	Cmax3	Cmax4	Average		Cmax1	Cmax2	Cmax3	Cmax4	Average
				mean	0.025687	0.041623	0.033578	0.028869	0.0324392	median	425.714	476.052	513.413	540.549	494.7325
				median	0.0258	0.0416	0.033537	0.028909	0.0312228	min	394.646	437.709	469.957	494.536	394.646
				min	0.017533	0.037004	0.027228	0.021361	0.017533	max	453.833	527.964	581.645	622.545	622.545
				max	0.032405	0.045648	0.039065	0.035142	0.045648	max-min	59.187	90.255	111.688	128.009	97.28475
4	validation	risperidone	inter	1.81	Cmax1	Cmax2	Cmax3	Cmax4	Average		Cmax1	Cmax2	Cmax3	Cmax4	Average
				mean	0.062752	0.061771	0.060975	0.060282	0.0614449	median	318.155	320.318	323.66	328.38	321.989
				median	0.062899	0.061869	0.06107	0.060333	0.0614693	min	307.536	307.536	305.767	305.373	305.373
				min	0.057751	0.05616	0.055157	0.054375	0.054375	max	343.717	350.993	357.482	364.167	364.167
				max	0.064809	0.064837	0.064784	0.06472	0.064837	max-min	36.181	43.457	51.715	58.794	47.53675
5	validation	domperidone	inter	19	Cmax1	Cmax2	Cmax3	Cmax4	Average		Cmax1	Cmax2	Cmax3	Cmax4	Average
				mean	0.06297	0.059898	0.057309	0.0551	0.0588194	median	330.543	351.583	365.15	378.915	358.3665
				median	0.062879	0.059786	0.05718	0.054991	0.0584828	min	307.536	316.188	326.807	335.459	307.536
				min	0.059113	0.055658	0.052976	0.050829	0.050829	max	350.993	374.589	391.303	406.444	406.444
				max	0.067835	0.065413	0.063107	0.061205	0.067835	max-min	43.457	58.401	64.496	70.985	59.33475
6	validation	clarithromycin	inter	1206	Cmax1	Cmax2	Cmax3	Cmax4	Average		Cmax1	Cmax2	Cmax3	Cmax4	Average
				mean	0.062995	0.062163	0.061502	0.060934	0.0618985	median	315.402	318.942	323.661	328.38	321.3015
				median	0.063062	0.062214	0.061558	0.060981	0.061886	min	307.733	308.717	310.88	313.043	307.733
				min	0.061283	0.059949	0.058927	0.058072	0.058072	max	328.183	334.279	341.555	346.274	346.274
				max	0.064744	0.064625	0.064458	0.064316	0.064744	max-min	20.45	25.562	30.675	33.231	27.4795
7	validation	astemizole	inter	0.26	Cmax1	Cmax2	Cmax3	Cmax4	Average		Cmax1	Cmax2	Cmax3	Cmax4	Average
				mean	0.059275	0.057204	0.055685	0.054455	0.0566546	median	330.018	340.638	348.702	353.815	344.67
				median	0.059267	0.057245	0.055676	0.054449	0.0564603	min	317.234	322.741	328.248	331.591	317.234
				min	0.055133	0.052848	0.051333	0.050122	0.050122	max	350.472	363.649	371.712	376.826	376.826
				max	0.06257	0.061105	0.059827	0.058923	0.06257	max-min	33.238	40.908	43.464	45.235	40.71125
8	validation	droperidol	inter	6.33	Cmax1	Cmax2	Cmax3	Cmax4	Average		Cmax1	Cmax2	Cmax3	Cmax4	Average
				mean	0.055192	0.051062	0.047409	0.059804	0.0533668	median	327.2	349.223	369.28	386.977	359.2515
				median	0.055224	0.051013	0.047351	0.059872	0.0531183	min	316.975	328.773	343.718	358.858	316.975
				min	0.050234	0.045988	0.042613	0.055755	0.042613	max	360.431	387.567	409.394	425.321	425.321
				max	0.05961	0.056432	0.053287	0.062526	0.062526	max-min	43.456	58.794	65.676	66.463	58.59725
9	validation	pimozide	inter	0.431	Cmax1	Cmax2	Cmax3	Cmax4	Average		Cmax1	Cmax2	Cmax3	Cmax4	Average
				mean	0.064149	0.063547	0.062995	0.062479	0.0632924	median	311.862	316.188	319.531	321.694	317.8595
				median	0.064204	0.063583	0.063039	0.06252	0.0633108	min	304.194	304.194	304.194	306.357	304.194
				min	0.061449	0.060213	0.059298	0.058543	0.058543	max	324.644	331.526	337.425	342.144	342.144
				max	0.066772	0.066864	0.06681	0.066643	0.066864	max-min	20.45	27.332	33.231	35.787	29.2
10	validation	clozapine	inter	71	Cmax1	Cmax2	Cmax3	Cmax4	Average		Cmax1	Cmax2	Cmax3	Cmax4	Average
				mean	0.060309	0.059088	0.058046	0.061836	0.0598198	median	319.531	326.413	332.509	337.228	329.461
				median	0.060356	0.059131	0.058108	0.061864	0.0597435	min	311.862	316.188	317.171	319.334	311.862
				min	0.057878	0.056184	0.054791	0.060031	0.054791	max	329.756	336.638	345.29	352.566	352.566
				max	0.062281	0.061318	0.060512	0.063348	0.063348	max-min	17.894	20.45	28.119	33.232	24.92375
11	validation	metoprolol	low	1800	Cmax1	Cmax2	Cmax3	Cmax4	Average		Cmax1	Cmax2	Cmax3	Cmax4	Average
				mean	0.058032	0.053927	0.050592	0.047752	0.0525757	median	330.347	346.471	356.499	369.674	351.485
				median	0.05802	0.05385	0.050574	0.047792	0.052212	min	315.009	326.021	336.049	349.224	315.009
				min	0.053864	0.048808	0.045111	0.04214	0.04214	max	343.128	364.365	378.129	392.68	392.68
				max	0.061488	0.058078	0.054871	0.052159	0.061488	max-min	28.119	38.344	42.08	43.456	37.99975
12	validation	tamoxifen	low	21	Cmax1	Cmax2	Cmax3	Cmax4	Average		Cmax1	Cmax2	Cmax3	Cmax4	Average
				mean	0.063938	0.063542	0.063199	0.062888	0.0633917	median	309.896	312.453	315.009	316.189	313.731
				median	0.063969	0.063562	0.06321	0.062897	0.0633858	min	307.34	307.34	307.34	308.52	307.34
				min	0.062611	0.06189	0.061262	0.060722	0.060722	max	317.565	322.678	325.234	328.97	328.97
				max	0.064838	0.064887	0.064891	0.064884	0.064891	max-min	10.225	15.338	17.894	20.45	15.97675
13	validation	loratadine	low	0.45	Cmax1	Cmax2	Cmax3	Cmax4	Average		Cmax1	Cmax2	Cmax3	Cmax4	Average
				mean	0.064784	0.064885	0.064964	0.06503	0.0649156	median	307.2	307.2	307.2	307.2	307.2
				median	0.064789	0.064887	0.064959	0.065023	0.0649228	min	307.201	307.201	307.201	307.201	307.201
				min	0.064506	0.064499	0.064498	0.064499	0.064498	max	307.202	307.202	307.202	307.202	307.202
				max	0.065069	0.065241	0.065397	0.065507	0.065507	max-min	0.001	0.001	0.001	0.001	0.001
14	validation	nifedipine	low	7.7	Cmax1	Cmax2	Cmax3	Cmax4	Average		Cmax1	Cmax2	Cmax3	Cmax4	Average
				mean	0.075559	0.077225	0.078423	0.07291	0.0760292	median	293.575	287.873	283.547	285.12	286.4965
				median	0.075603	0.07723	0.07841	0.072922	0.0764163	min	288.07	282.76	278.434	277.451	277.451
				min	0.073134	0.074567	0.075626	0.070998	0.070998	max	298.688	292.985	288.659	287.676	298.688
				max	0.077292	0.079762	0.081456	0.074987	0.081456	max-min	10.618	10.225	10.225	10.225	10.32325
15	validation	nitrendipine	low	3.02	Cmax1	Cmax2	Cmax3	Cmax4	Average		Cmax1	Cmax2	Cmax3	Cmax4	Average
				mean	0.06995	0.067709	0.068726	0.069418	0.0689506	median	303.211	301.048	299.081	300.458	300.753
				median	0.070033	0.067805	0.068845	0.069514	0.069179	min	295.542	293.379	288.856	290.233	288.856
				min	0.064284	0.063723	0.063859	0.064071	0.063723	max	321.104	321.498	322.087	323.464	323.464
				max	0.073508	0.070719	0.072077	0.072889	0.073508	max-min	25.562	28.119	33.231	33.231	30.03575
